# Thymol Impacts the Progression of Endometriosis by Disrupting Estrogen Signaling Pathways and Inflammatory Responses

**DOI:** 10.3390/ijms252313150

**Published:** 2024-12-07

**Authors:** Yu Zhang, Aftab Shaukat, Han Zhang, Yao-Feng Yang, Hui-Xia Li, Guang-Ya Li, Ying-Nan Liu, Chen Liang, Jin-Wen Kang, Shao-Chuan Li, Ren-Wei Su

**Affiliations:** 1 College of Veterinary Medicine, South China Agricultural University, Guangzhou 540642, China; 20231027017@stu.scau.edu.cn (Y.Z.); dr.aftabshaukat@scau.edu.cn (A.S.); 20222027044@stu.scau.edu.cn (H.Z.); 1271067857@stu.scau.edu.cn (Y.-F.Y.); lihuixia@stu.scau.edu.cn (H.-X.L.); 13545080045@stu.scau.edu.cn (G.-Y.L.); liuyingnan0902@163.com (Y.-N.L.); 20221027009@stu.scau.edu.cn (C.L.); jwkang2020@163.com (J.-W.K.); 2 Key Laboratory of Animal Vaccine Development, Ministry of Agriculture, Guangzhou 510642, China

**Keywords:** endometriosis, thymol, estrogen signaling, inflammation

## Abstract

Endometriosis is a chronic inflammatory, estrogenic disorder caused by endometrial tissue growth places other than uterine lumen, resulting in infertility and severe pelvic pain. Thymol, an extract of *Thymus vulgaris*, processes diverse biological properties, including anti-inflammatory, local anesthetic, decongestant, and antiseptic effects. However, the efficacy of thymol in treating endometriosis has still not been explored. Herein, this research aimed to investigate the role of thymol in the treatment of endometriosis using a murine model and Ishikawa cells. Thirty C57BL/6 mice were administered 17β-E2 (100 ng/mouse) subcutaneously for three consecutive days to induce synchronous estrus. On the last day of injection, the mice underwent surgical induction of endometriosis. After that, the mice were divided into three groups, i.e., Control (CTRL), Thymol 30 mg/kg and Thymol 60 mg/kg, receiving oral administration of either saline or thymol (30 mg/kg/d or 60 mg/kg/d, as 0.1 mL/kg/d, respectively) for a three-week duration. Each group consisted of ten mice and was evenly divided into estrus and diestrus according to the vaginal cytology on the last day of treatment. Thymol significantly (*p* < 0.05) reduced the weight and volume of ectopic tissue, hindered cell proliferation, and stimulated apoptosis compared to the CTRL group. Additionally, in the thymol-treated group, the levels of pro-inflammatory cytokines, *tumor necrosis factor-α (TNF-α)*, *interleukin (IL)-1β*, and *IL-6*, as well as the numbers of neutrophils and macrophages, were significantly (*p* < 0.05) decreased. Moreover, a novel role of thymol in rebalancing estrogen and progesterone (E_2_-P_4_) signaling was explored, and it was distributed in the ectopic endometrium. Next, the role of thymol on Ishikawa cells was determined. The results demonstrated that thymol significantly (*p* < 0.05) suppressed the E_2_-induced proliferation of Ishikawa cells. Furthermore, molecular docking analyses suggested that thymol potentially binds to ESR1-like estrogens, indicating its antagonistic activity against estrogens. The estrogen receptor 1 (ESR1) and its target gene expression exhibited significant (*p* < 0.05) downregulation, while progesterone receptor (PGR) and target genes were markedly (*p* < 0.05) upregulated following thymol treatment in the ectopic endometrium. Most importantly, our data revealed the minimal impact of thymol treatment on the eutopic endometrium and its crucial role in supporting pregnancy, thus indicating the safety of thymol in treating endometriosis. Overall, our study suggests that thymol holds promising therapeutic implications for endometriosis by virtue of its anti-inflammatory properties and ability to antagonize estrogen activity.

## 1. Introduction

Endometriosis is a chronic inflammatory disorder that depends on estrogen, characterized by the existence and growth of endometrial cells on the exterior of the uterus, referred to as ectopic endometrium, impacting 10–15% of women of reproductive age [1,2]. Typical manifestations of endometriosis are persistent discomfort, exhaustion, and infertility [3]. About 25 to 50% of infertile women have endometriosis, and 30 to 50% of women with endometriosis are infertile [3]. Endometriosis can interfere with normal functioning of the bowel and/or bladder, depending on the site where endometriotic lesions develop [4]. Endometriosis also impacts mental health, with one study showing that 87% of the women investigated with endometriosis had depressive symptoms and 88% had anxiety [5]. Factors influencing the pathogenesis of endometriosis include hormonal imbalances, immune dysfunction, genetic predisposition, and oxidative stress in the peritoneal cavity [6,7,8,9]. Among theories regarding endometriosis pathogenesis, Sampson’s retrograde menstrual implantation theory has achieved the most acceptance. The retrograde menstruation theory is the predominant explanation for endometriotic pathogenesis, positing that endometrial debris flows backward through the fallopian tubes into the peritoneal cavity, then adheres to and infiltrates the pelvic mesothelium and other pelvic organs, ultimately forming endometriotic lesions [10].

Estrogen and progesterone play vital roles in female reproduction through their nuclear receptors: estrogen receptors (ESRs) and progesterone receptors (PGRs), respectively. Estrogen, particularly 17β-estradiol (E_2_), induces PGR expression via ESRs, and the ensuing activation of progesterone signaling reciprocally suppresses estrogen signaling, establishing a finely tuned feedback system that balances downstream effects [11]. When the estrogen and progesterone (E_2_–P_4_) balance is disrupted, various gynecological disorders, including endometriosis, can arise. In ectopic endometrium, a higher level of ESR1, activated by ovarian and locally synthesized E_2_, leads to excessive proliferation of endometrial tissues [12,13]. Contrastingly, the response of endometrial tissue, especially in ectopic endometrium, to progesterone is impaired due to the decrease in expression of PGR and its co-activators through transcriptional and epigenetic modifications, resulting in a phenomenon called progesterone resistance, inhibiting the role of progesterone in balancing estrogen-induced proliferation [14]. Another estrogen receptor, ESR2, is upregulated in women with endometriosis and has been shown to drive the pathogenesis of endometriosis via modulating apoptosis complexes and the inflammasome in the transgenic mouse model [15,16].

Immunological dysfunction significantly contributes to the development of endometriosis, particularly within the pelvic cavity, where the majority of lesions are located. Macrophages, the tissue-resident cells that bridge innate and adaptive immunity, have a pivotal role in maintaining local homeostasis in a healthy state and in the development and persistence of various inflammatory conditions [17]. Macrophages accumulate in the peritoneal cavity of women with endometriosis due to the local production of chemotactic chemicals [18]. Nonetheless, peritoneal macrophages can eradicate endometrial tissue under typical circumstances, and this phagocytic mechanism seems ineffective in endometriosis [19]. The peritoneal lining consists of monolayer epithelioid cells, called mesothelial cells (MCs), of mediator origin. Upon exposure to leukocytes, they produce pro-inflammatory mediators; activated MCs produce numerous biologically active molecules, including lipid mediators (prostaglandins and prostaglandins), growth factors, cytokines, chemokines, and adhesion molecules, resulting in the recruitment of additional leukocytes to the mesothelium [20,21].

Hormone therapy and surgical treatment are the prevailing approaches for managing endometriosis [22]. Non-steroidal anti-inflammatory drugs (NSAIDs) are also used alongside hormonal and surgical treatments [23]. Nevertheless, recent studies have indicated a link between NSAID use and an increased risk of thrombotic events [24], and NSAID can have side effects on the gastrointestinal tract [25]. Natural plant products are abundant in biologically active compounds. Recently, there has been increasing interest in using plant-derived drugs for treating endometriosis [26]. It has been reported that the prunus flavonoid compound prunuses can inhibit the growth of endometriosis both in vivo and in vitro by downregulating Cyclin E1 [27]. Another naturally occurring polyphenol, resveratrol, produced from grapes and legumes, has been found to inhibit endometriosis by boosting apoptosis and decreasing the proliferation of the endometrial cells [28].

Thyme has a long history of traditional use for therapeutic purposes, including treating illnesses related to the respiratory, digestive, circulatory, and nervous systems [29]. Thymol and the extract obtained from thyme essential oil are widely utilized for multiple purposes, such as medical antiseptics and wound healing agents, food preservatives, and flavorings [30,31,32]. Its pharmacological properties include antioxidant [33], anti-inflammatory [34], and antibacterial effects [35]. Thymol attenuates LPS-induced structural liver injury by reducing inflammatory cell infiltration, therefore protecting liver structure [36]. Thymol can also diminish the progression of airway hyper-responsiveness and inhibit the activation of the NF-κB pathway [37]. Thymol demonstrates antioxidant properties and may impede the advancement of hyperlipidemia and atherosclerosis generated by a high-fat diet by diminishing aortic intimal lipid lesions, decreasing blood lipid levels and oxidative stress, and mitigating inflammation-related reactions [37]. However, the role of thymol in the growth of endometriosis needs to be investigated. Therefore, the current study was planned to explore the beneficial protective effects of thymol on endometriosis using an endometriotic mouse model and Ishikawa cells. We treated the mouse model and Ishikawa cells with thymol and observed the growth of ectopic lesions and proliferation of cells, and detected the expression of inflammatory factors. Notably our findings might provide future insights for therapeutic strategies of endometriosis using thymol.

## 2. Results

### 2.1. Thymol Impedes Endometriotic Lesion Growth by Affecting Cell Proliferation and Apoptosis

**Figure 1:**
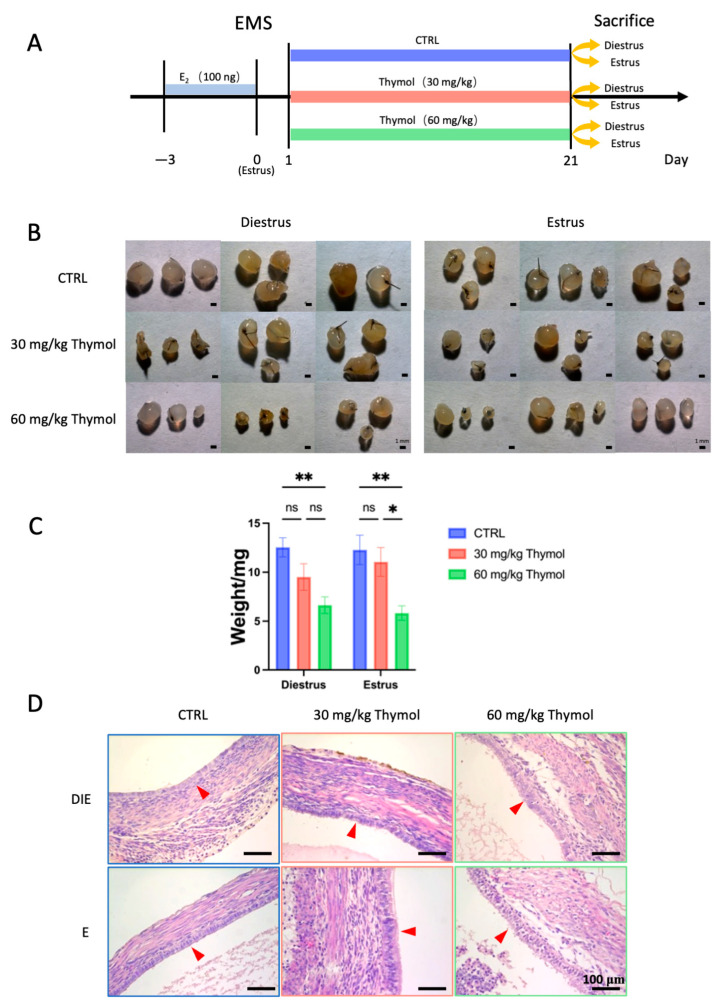
Growth inhibitory effect of thymol on endometriosis. (**A**) Illustration of the methodology employed in the endometriosis mice model treated with thymol. (**B**) The ectopic endometriosis lesion was photographed 21 days after treatment; each image was obtained from an individual mouse (*n* = 5). The scale bar is 1 mm. (**C**) The relative weight of lesions during the estrous and diestrous phases. (**D**) Histopathology of ectopic lesions (*n* = 5). Red arrowheads pointing epithelium cells. The scale bar is 100 µm. CTRL, DIE, E, E2, and EMS represent control, diestrus, estrus, estrogen, and endometriosis, respectively. The results are depicted as means ± SEM. ns represents non-significant results; * *p* < 0.05 and ** *p* < 0.01.

To establish an endometriosis model in mice, we surgically sutured the uterine fragments to the peritoneum (Figure 1A). A total of 30 mice were first randomly divided into three groups (CTRL, Thymol 30 mg/kg, and Thymol 60 mg/kg; 10 mice in each group). Since the growth of endometriotic lesions exhibits cyclical changes in an estrus cycle dependent upon estrogen concentration, we performed vaginal cytology for monitoring the estrus cycle after 21 days of treatment. We then divided the 10 mice of each group into estrus and diestrus stages accordingly (*n* = 5 for each sub-group). Our findings showed that thymol markedly (*p* < 0.05) decreased the weight and size of ectopic tissues in both the estrus and diestrus, showing a dose-dependent response and indicating the influential roles of thymol in inhibiting the ectopic tissue growth (Figure 1B,C). Histological staining revealed that the epithelium of ectopic tissues in the CTRL group exhibited cuboidal morphology, whereas those from the Thymol-treated groups displayed a more columnar appearance (Figure 1D).

**Figure 2:**
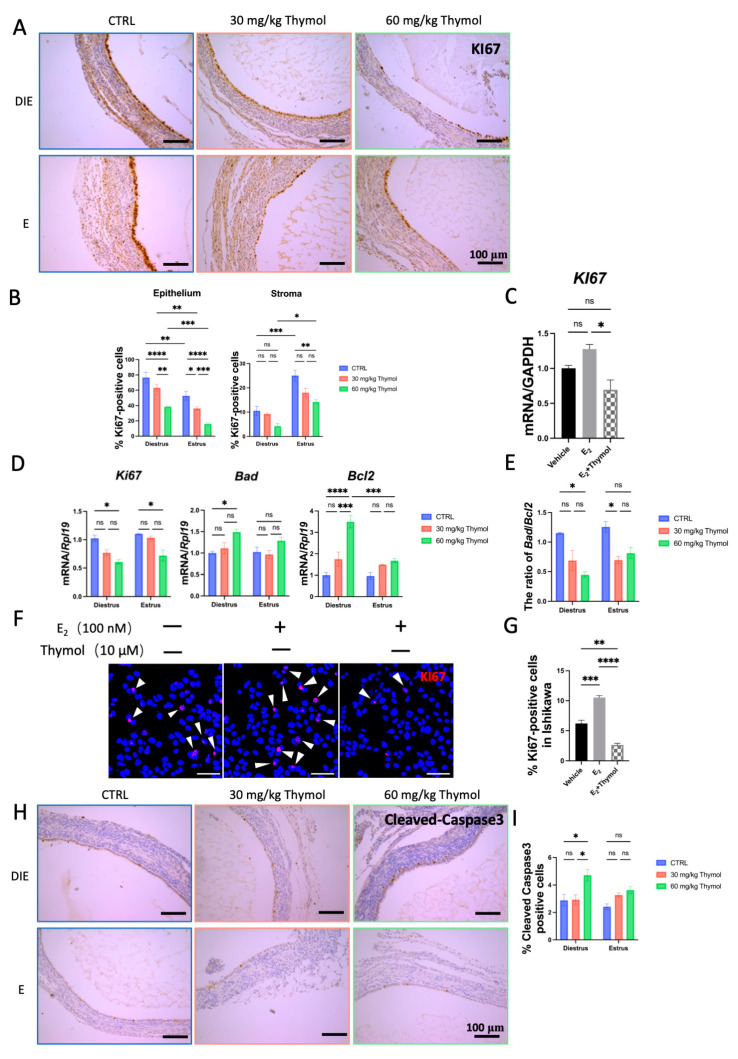
Thymol hinders cell proliferation and promotes cell apoptosis. (**A**) Ki67 immunostaining of ectopic lesions (*n* = 5); 100 µm scale bar was employed. (**B**) Ki67-positive cell percentage in CTRL and Thymol groups during estrus and diestrus. (**C**) The relative mRNA expression of *Ki67* in Ishikawa cells. (**D**) The relative mRNA expression of *Ki67*, *Bad*, and *Bcl2* in CTRL and Thymol groups during estrus and diestrus (*n* = 5). (**E**) The ratio of the relative mRNA expression of *Bad*/ *Bcl2*. (**F**) Ki67 immunofluorescence in Ishikawa cells. White arrowheads point to Ki67-positive cells. (**G**) Ki67-positive cell percentage in Ishikawa cells after thymol and/or E2 treatment. (**H**) Cleaved-Caspase-3 immunostaining of ectopic lesions (*n* = 5); 100 µm scale bar was employed. (**I**) Cleaved-Caspase-3-positive cell percentage in CTRL and Thymol groups during estrus and diestrus. DIE, E E2, and CTRL represent diestrus, estrus, estrogen, and control groups, respectively. The results are depicted as means ± SEM. ns represents non-significant results; * *p* < 0.05, ** *p* < 0.01, *** *p* < 0.001, and **** *p* < 0.0001.

Subsequently, we investigated the proliferation and apoptosis of ectopic tissues. Ki67 staining revealed that thymol significantly reduced (*p* < 0.05) the growth and proliferation of the ectopic epithelial cells, regardless of the estrus status (Figure 2A). However, the inhibitory effect of thymol on the ectopic stroma during diestrus was less prominent compared to that during estrus, partly due to the lower level of stromal proliferation in diestrus (Figure 2B,D,E). Next, we explored the role of thymol on the human endometrial epithelium (Ishikawa cells) using immunofluorescence and qPCR. To select the appropriate experimental dose, we first performed a cytotoxicity test for thymol on the Ishikawa cells using the CCK8 assay (Figure S1C). Consequently, a 10 μM dose of thymol was chosen for further study. The results demonstrated that thymol significantly suppressed the E_2_-induced proliferation of Ishikawa cells (Figure 2C,F,G). On the other hand, Cleaved-Caspase3 staining exhibited elevated levels in ectopic tissues, indicating increased apoptosis during diestrus but not during the estrus phase due to thymol treatment (Figure 2H,I). Altogether, our results suggested that the reduction in ectopic tissue growth by thymol treatment can be attributed to its combined effects on cell proliferation and apoptosis.

### 2.2. Thymol Mitigates the Inflammatory Reaction in Endometriotic Lesions

**Figure 3:**
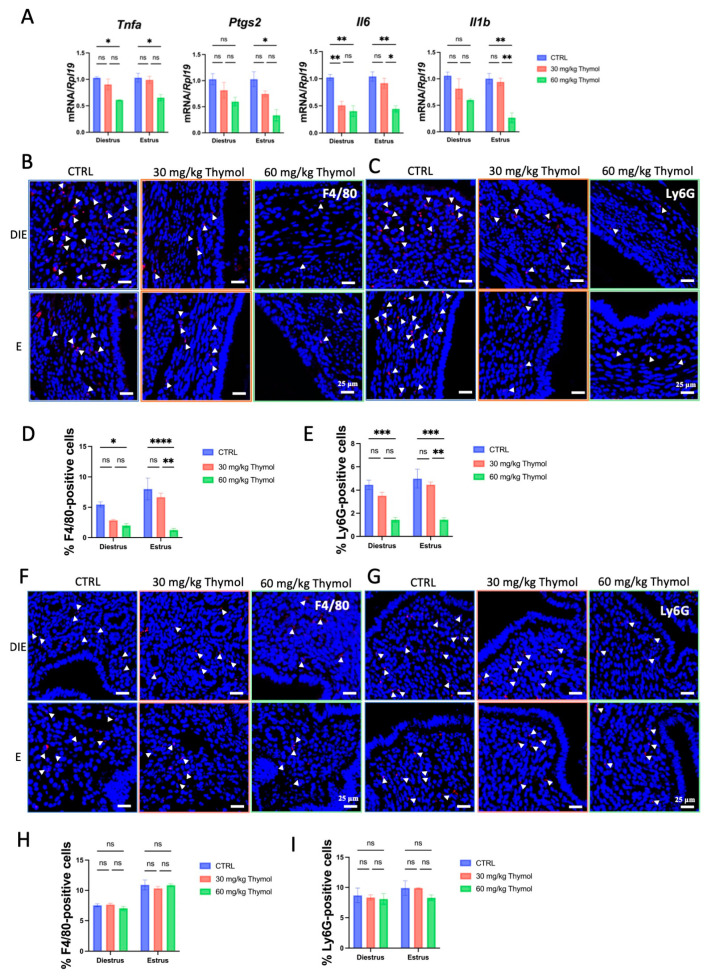
Suppressive inflammatory effect of thymol in endometriotic tissue. (**A**) Relative mRNA expression of *Tnfa*, *Ptgs2*, *Il1b*, and *Il6* in lesions of CTRL and Thymol groups (*n* = 5). (**B**) F4/80 immunofluorescence in the ectopic lesion of CTRL and Thymol groups during estrus and diestrus (*n* = 5); White arrowheads point to F4/80-positive cells, 25 µm scale bar was employed. (**C**) Ly6G immunofluorescence in the ectopic lesion of CTRL and Thymol groups during estrus and diestrus (*n* = 5) White arrowheads point to Ly6G-positive cells. (**D**) F4/80-positive cell percentage in CTRL and Thymol groups during estrus and diestrus. (**E**) Ly6G-positive cell percentage in CTRL and Thymol groups during estrus and diestrus; 25 µm scale bar was employed. (**F**) F4/80 immunofluorescence in ectopic uteri of CTRL and Thymol groups during estrus and diestrus (*n* = 5); White arrowheads point to F4/80-positive, 25 µm scale bar was employed. (**G**) Ly6G immunofluorescence in ectopic uteri of CTRL and Thymol groups during estrus and diestrus (*n* = 5); White arrowheads point to Ly6G-positive cells, 25 µm scale bar was employed. (**H**) F4/80-positive cell percentage in ectopic uteri of CTRL and Thymol groups during estrus and diestrus. (**I**) Ly6G-positive cell percentage in ectopic uteri of CTRL and Thymol groups during estrus and diestrus (*n* = 5). DIE, E, and CTRL represent diestrus, estrus, and control groups, respectively. The results are depicted as means ± SEM. ns represents non-significant results; * *p* < 0.05, ** *p* < 0.01, *** *p* < 0.001, and **** *p* < 0.0001.

An inflammatory environment facilitates endometriosis. We evaluated the impact of thymol on the local inflammatory response in ectopic tissues by analyzing the production of critical inflammatory cytokines. Pro-inflammatory markers’ mRNA expression was markedly (*p* < 0.05) reduced in the ectopic tissues of estrous-phase mice administered 60 mg/kg of thymol, in comparison to the CTRL group (Figure 3A). In diestrus mice, both high and low doses of thymol led to downregulation of *Il6*. In contrast, *Tnfa* expression was only affected by the high dose (Figure 3A). Additionally, the high dose of thymol inhibited the expression of the *Prostaglandin-Endoperoxide Synthase 2* (*Ptgs2*) gene, which encodes Cyclooxygenase-2 (COX2) in the estrus phase (Figure 3A). To further explore the effects of thymol on inflammation, we assessed the presence of immune cells, specifically neutrophils (Ly6G) and macrophages (F4/80), in ectopic tissues. The findings of immunofluorescence proved that a substantial decrease in the number of macrophages (Figure 3B,D) and neutrophils (Figure 3C,E) was significantly (*p* < 0.05) reduced in ectopic tissues of thymol-treated mice. However, the counts of macrophages (Figure 3F,H) and neutrophils (Figure 3G,I) in the eutopic endometrium was not affected during either the diestrus or estrus phases. These results suggest that thymol effectively suppressed the inflammatory response in ectopic endometriosis while not affecting eutopic endometrium.

### 2.3. Thymol Rebalances Estrogen–Progestogen Signaling in Ectopic Tissue

The imbalance between progesterone and estrogen has a vital role in the progression of endometriosis, where the ectopic tissue becomes more responsive to estrogen but develops resistance to progesterone treatment [38]. Hence, we investigated whether thymol could modulate the activation of the hormone signaling of these two domains.

**Figure 4:**
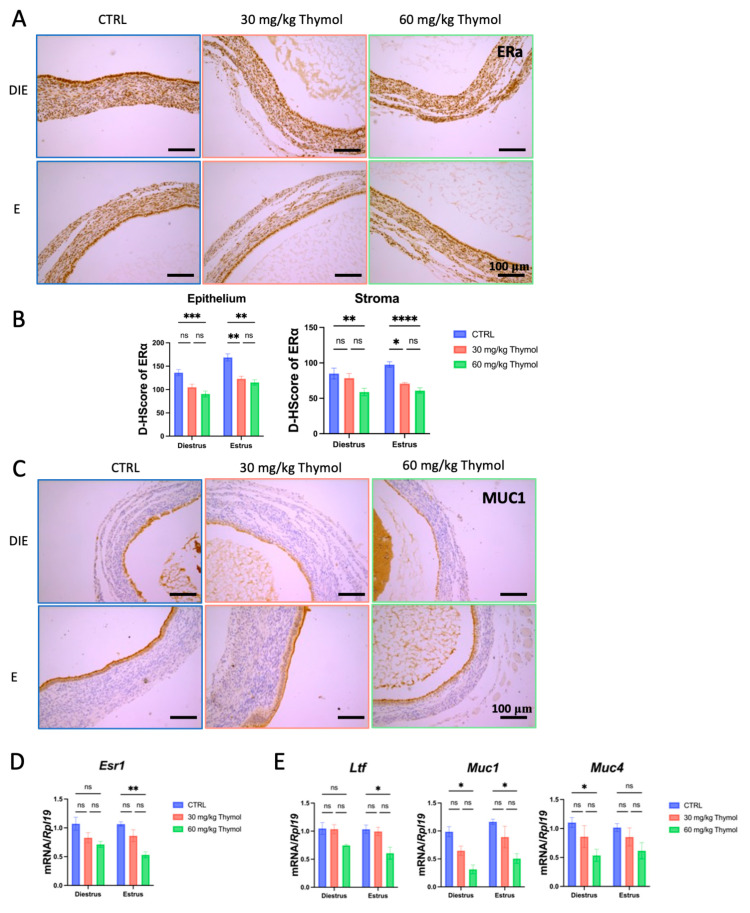
Thymol interferes with estrogen signaling pathways. (**A**) ESR1 immunostaining of ectopic lesions (*n* = 5); 100 µm scale bar was employed. (**B**) ESR1 H-Score expression both in stromal and epithelial cells. (**C**) MUC1 immunostaining of ectopic lesions (*n* = 5); 100 µm scale bar was employed. (**D**) The mRNA levels of *Esr1* in ectopic lesions of CTRL and thymol-treated mice (*n* = 5). (**E**) Relative expression of *Muc1*, *Muc4*, and *Ltf* in endometriosis lesions (*n* = 5). DIE, E, and CTRL represent diestrus, estrus, and control groups, respectively. The results are depicted as means ± SEM. ns represents non-significant results; * *p* < 0.05, ** *p* < 0.01, *** *p* < 0.001, and **** *p* < 0.0001.

To assess whether thymol hindered the growth of endometriotic ectopic tissues by influencing estrogen and progesterone signaling, we examined the expression level of progesterone and estrogen receptors as well as their target genes in the ectopic tissue of CTRL and thymol-treated mice. ESR1 exhibited predominant expression in both the luminal epithelium and stroma, which was reduced following thymol treatment during both the estrus and diestrus phases (Figure 4A,B). Similarly, the expression of the mucin (MUC)1 protein was inhibited by thymol in both the diestrous and estrous phases (Figure 4C). At the mRNA level, thymol downregulated *Esr1* and its targets genes, including *Muc1, Muc4*, and *lactotransferrin* (*Ltf*), in ectopic tissues (Figure 4D,E).

**Figure 5:**
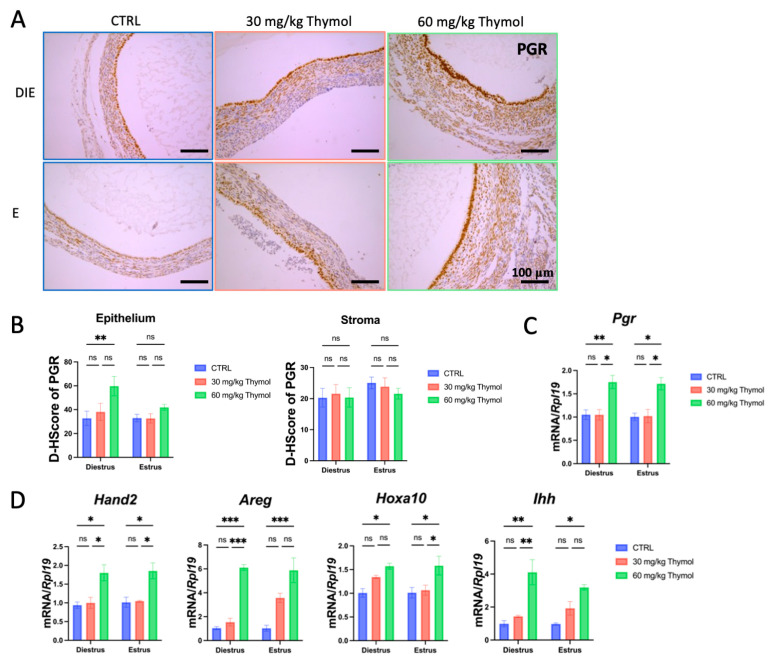
Thymol interferes with progesterone signaling pathways. (**A**) PGR immunostaining of ectopic lesions (*n* = 5); 100 µm scale bar was employed. (**B**) PGR H-Score expression is present in both stromal and epithelial cells of ectopic tissue. (**C**) The mRNA levels of *Pgr* in ectopic lesions of CTRL and thymol-treated mice (*n* = 5). (**D**) Relative expression of *Hand2*, *Hoxa10*, *Ihh*, and *Areg* in endometriosis lesions (*n* = 5). DIE, E, and CTRL represent diestrus, estrus, and control groups, respectively. The results are depicted as means ± SEM. ns represents non-significant results; * *p* < 0.05, ** *p* < 0.01, and *** *p* < 0.001.

On the other hand, thymol also interfered with the progesterone signaling pathway. Immunostaining demonstrated an increase in the expression of PGR during diestrus, which was increased after thymol administration and restricted to the luminal epithelium during diestrus pause (Figure 5A,B). Moreover, thymol significantly (*p* < 0.05) upregulated the mRNA levels of *Pgr* and its target genes, including *heart and neural crest derivatives-expressed transcript 2* (*Hand2*)*, homeobox gene A10* (*Hoxa10*)*, Indian hedgehog* (*Ihh*)*,* and *Amphiregulin* (*Areg*) in ectopic tissues (Figure 5C,D). These findings indicate that thymol inhibition of ectopic lesion growth is associated with the rebalance of estrogen–progesterone signaling.

### 2.4. Thymol Acts as an Antagonist of Estrogen

**Figure 6:**
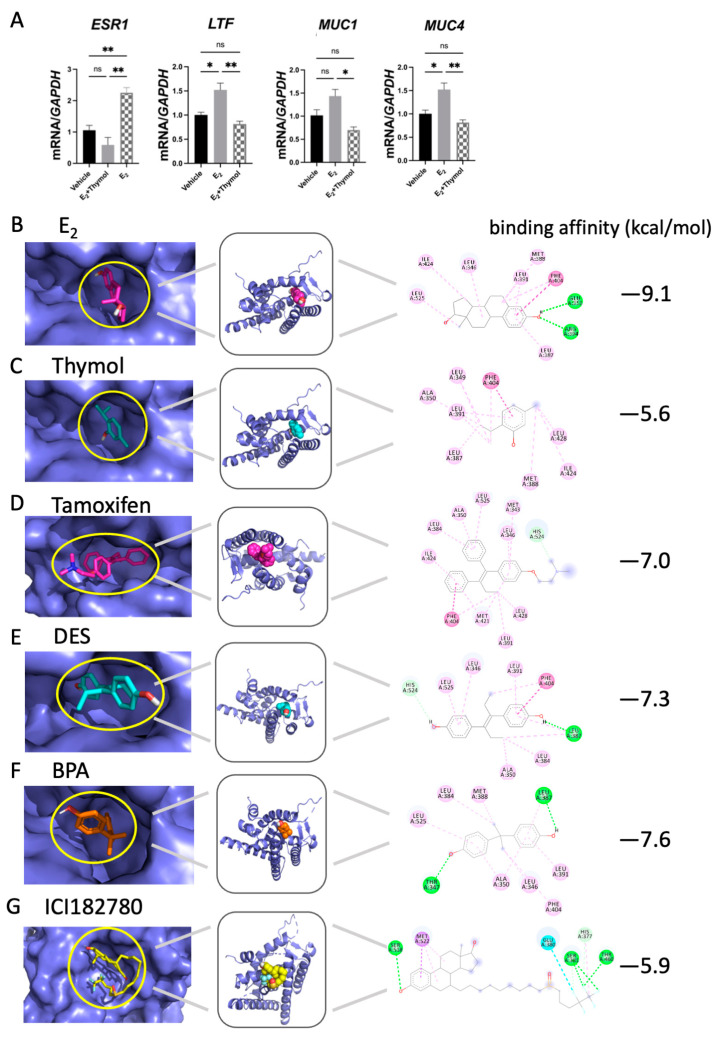
Thymol competitively bound ESR1 against estrogen. (**A**) The mRNA expression of *ESR1* and estrogen target genes (*LTF*, *MUC1*, and *MUC4*) in E2-treated Ishikawa cells (*n* = 5). (**B**) Prediction of binding modes of E2 with ESR1 protein using molecular modeling. (**C**) Prediction of binding modes of thymol with ESR1 protein using molecular modeling. (**D**) Prediction of binding modes of tamoxifen with ESR1 protein using molecular modeling. (**E**) Prediction of binding modes of BPA with ESR1 protein using molecular modeling. (**F**) Prediction of binding modes of DES with ESR1 protein using molecular modeling. (**G**) Prediction of binding modes of ICI182780 with ESR1 protein using molecular modeling. The results are depicted as means ± SEM. ns represents non-significant results; * *p* < 0.05 and ** *p* < 0.01.

Moreover, to determine whether the anti-estrogen effect of thymol in our endometriotic model was a direct outcome or simply attributable to slower growth of the ectopic lesion, we treated Ishikawa cells with 100 nM E_2_, either with or without 10 μM thymol, for a duration of 12 h. The results obtained from this in vitro experiment demonstrated significant suppression of *ESR1* and target genes *LTF*, *MUC1*, and *MUC4* by thymol treatment when induced by E_2_, providing strong evidence that thymol acts as an antagonist of E_2_ (Figure 6A).

Next, molecular docking analysis assessed the binding efficiency between ESR1 and E_2_, thymol, and other estrogen antagonists. The calculated average binding energies of E_2_, thymol, tamoxifen, DES, BPA, and ICI182780 with ESR1 were −9.1, −5.6, −7.0, −7.3, −7.6, and −5.9 kcal/mol, respectively (Figure 6B–G). According to the calculation, thymol displayed a similar ESR1-binding activity to ICI182720, a well-known estrogen antagonist, although weaker than the ESR1-binding activity of E_2_, tamoxifen, DES, and BPA. To gain further insights into the potential mechanism of thymol, computational analysis through molecular docking analysis was used, revealing that thymol has the potential to bind to the groove on the surface of the ESR1 protein, with a binding with PEP404 considered as a potential binding site (Figure 6B). Accordingly, E_2_ forms hydrogen bonds with ARG394 and GLU353 of ESR1 proteins (Figure 6A). These outcomes suggest that thymol functions as an estrogen antagonist in endometriosis treatment, likely by competitively binding to ESR1, thereby impeding the binding of E_2_ to the protein, and consequently obstructing the development of endometriosis.

### 2.5. Thymol Affects Estrogen and Progesterone Signaling in Eutopic Endometrium Without Impacting the Establishment of Pregnancy

**Figure 7:**
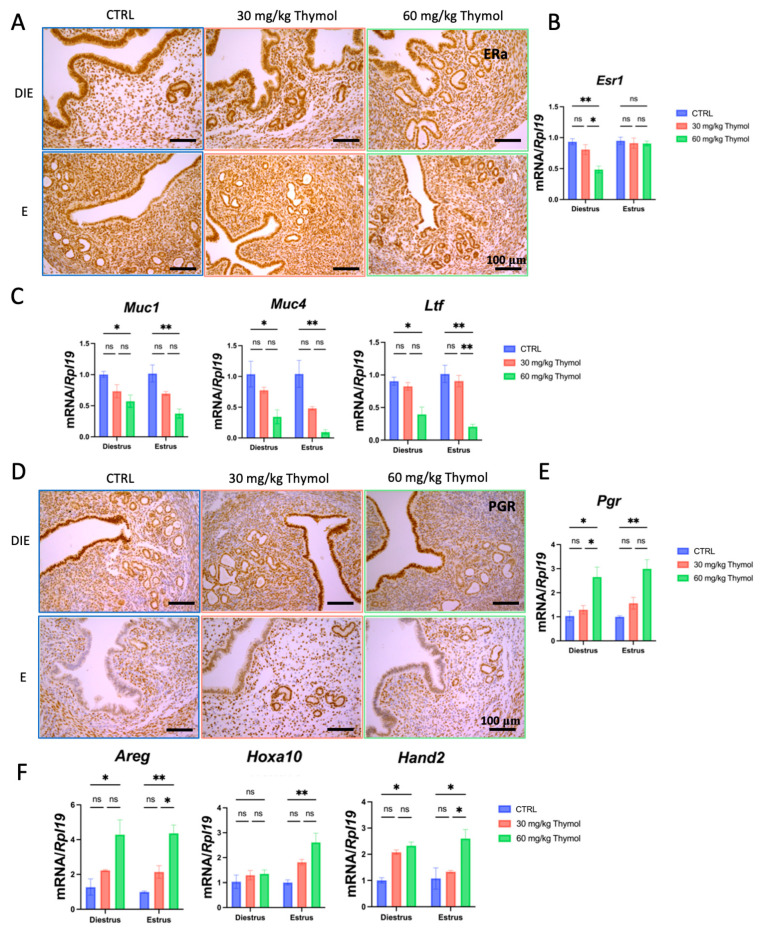
The influence of thymol on eutopic uteri in mice. (**A**) ESR1 immunostaining of eutopic uteri (*n* = 5); 100 µm scale bar was employed. (**B**) The mRNA levels of *Esr1* in eutopic uteri of CTRL and thymol-treated mice (*n* = 5). (**C**) Relative expression of *Muc1*, *Muc4*, and *Ltf* in endometriosis lesions (*n* = 5). (**D**) PGR immunostaining of eutopic uteri (*n* = 5); 100 µm scale bar was employed. (**E**) The mRNA levels of *Pgr* in eutopic uteri of CTRL and thymol-treated mice (*n* = 5). (**F**) Relative expression of *Hand2*, *Hoxa10*, *Ihh*, and *Areg* in endometriosis lesions (*n* = 5). DIE, E, and CTRL represent diestrus, estrus, and control groups, respectively. The results are depicted as means ± SEM. ns represents non-significant results; * *p* < 0.05 and ** *p* < 0.01.

As a gynecological disease, infertility stands as a notable detrimental effect of endometriosis. In ART clinic, up to 50% of women with endometriosis suffer from infertility. Therefore, an endometriosis treatment that does not impact the normal functioning of the eutopic endometrium, especially the establishment of pregnancy, would be highly advantageous. Thus, we first focused on assessing the effects of thymol on the remaining eutopic uterine horn after removing the other horn to induce endometriosis. The results revealed that both a high and low dose of thymol slightly suppressed endometrial cell proliferation during diestrus, whereas only a high dose exhibited efficacy during estrus phases (Figure S1A). Notably, in diestrus, expression of ESR1 and its associated targets was reduced during both diestrus and estrus within the Thymol-treated groups as compared with the CTRL group (Figure 7A–C). Conversely, the expression of PGR and its targets significantly increased (Figure 7D–F). These observations indicate that thymol treatment influenced the eutopic endometrium, albeit to a lesser extent than its effects on the ectopic lesions.

**Figure 8:**
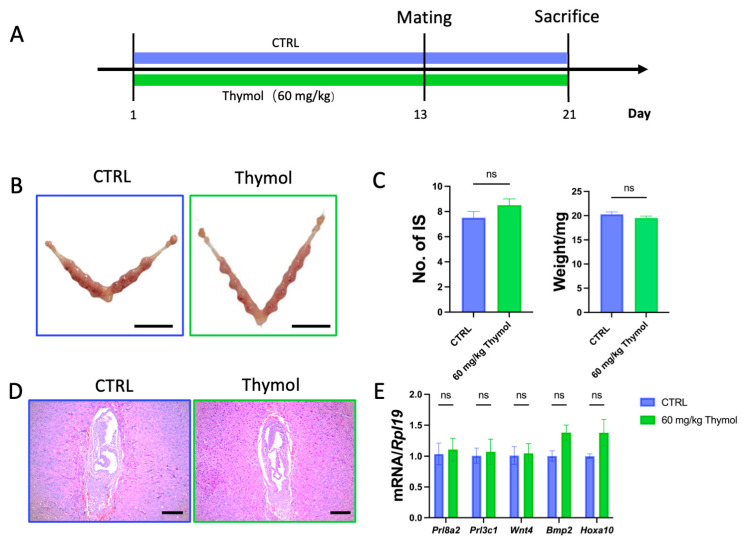
Thymol does not affect the fertility of mice. (**A**) Flow diagram of pregnancy induction and thymol treatment in mice. (**B**) Implantation sites of CTRL and Thymol group at 7.5 dpc; 1 cm scale bar was employed. (**C**) The number and the weight of implantation sites at 7.5 dpc in CTRL- and Thymol-group mice. (**D**) Histopathology of implantation sites at 7.5 dpc (*n* = 3); 100 µm scale bar was employed. (**E**) Relative expression of *Bmp2*, *Wnt4*, *Hoxa10*, *Prl8a2*, and *Prl3c1* in implantation sites (*n* = 3). CTRL and Thymol represent the implantation site of control group and Thymol-treated group, respectively. The results are depicted as means ± SEM. ns represents non-significant results.

Subsequently, we sought to determine whether the administration of thymol impacted the major physiological function of uterine: the capability to receive embryo implantation. We administered the high dose of thymol (60 mg/kg/d) to another group of mice for three weeks. On the 13th day of thymol administration, the females were mated with fertile males to induce pregnancy. The next day was marked as postcoital day 0.5 (dpc 0.5) as a vaginal plug was observed. The thymol administration continued for one more week. At dpc 7.5, the 21st day of thymol treatment, the mice were sacrificed (Figure 8A). The results demonstrated no significant differences in the number and weight of implantation sites in thymol-treated mice at dpc 7.5 compared to the CTRL group mice (Figure 8B,C). The typical morphology of implantation sites was confirmed by H&E staining, as no apparent abnormalities were observed (Figure 8D). At dpc 7.5, the decidualization of endometrial stroma represents a crucial event during pregnancy. Therefore, we tested the expression of decidualization marker genes, including *Bone morphogenetic protein 2* (*Bmp2*)*, Wingless-related integration site 4* (*Wnt4*)*, Hoxa10, Prolactin Family8, Subfamily A, Member2* (*Prl8a2*)*,* and *Prolactin Family3, Subfamily C, Member1* (*Prl3c1*). The qPCR results illustrated no significant changes in the expression levels of these markers, suggesting a healthy pregnancy in the thymol-treated mice (Figure 8E). Overall, these findings indicate that the dosage and duration of endometriosis treatment we proposed had no detrimental effects on the reproductive performance of the mice.

## 3. Discussion

Endometriosis is recognized as a proliferative disease that is estrogen-dependent and is categorized by endometrial tissue growth outside the uterine cavity [39]. This abnormal growth is primarily attributed to increased cell proliferation and/or inhibited apoptosis in response to relevant stimuli [40]. Using a murine model of endometriosis, we examined the potential of thymol to mitigate the adverse effects of endometriosis in this study [16,41,42,43,44,45]. The results demonstrated that thymol inhibits the growth of endometriotic tissue by suppressing inflammatory pathways. Moreover, it was found that thymol acts as a novel estrogen antagonist by competitively binding to ESR1, which consequently inhibits the growth of endometriotic lesions. Furthermore, we confirmed the inhibitory effect of thymol on estrogen-induced proliferation using a human endometrial epithelial cell line. Importantly, this study provided evidence that the dosage of thymol treatment required to significantly inhibit ectopic endometrium growth is also safe for eutopic endometrium’s pregnancy function.

Endometriosis is a persistent inflammatory condition marked by an elevated presence of macrophages and their secretory substances [46]. The consistency of peritoneal fluid can be disrupted by an alteration in the cytokines produced by lymphocytes, which facilitates the growth of ectopic endometrial tissue [47]. The appropriate control and activation of macrophages and lymphocytes depend on a precise equilibrium of cytokine levels, which is disturbed in endometriosis [48]. Numerous investigations have shown that levels of IL-6 are markedly increased in endometriotic lesions, peritoneal fluid, and serum of women with endometriosis compared to disease-free controls [49,50,51,52]. Our study provides evidence of thymol’s ability to effectively reduce the population of macrophages and neutrophils and inhibit cytokine production. Consequently, thymol moderates local inflammatory response, which is consistent with its established anti-inflammatory function in other diseases [53,54].

Furthermore, our study has elucidated a previously unreported estrogen-antagonist mechanism by which thymol affects endometriosis. The suppressive effect of thymol on ectopic stromal tissue proliferation appeared to be significantly weaker during the diestrus compared to the estrus. This reduced efficacy may be attributed to the lower proliferation rates of stromal cells that is characteristic of diestrus. The hormone-dependent variation in stromal response underscores the importance of considering hormonal cycles when evaluating the potential therapeutic efficacy of thymol in endometriosis. Increased estrogen receptor activity, elevated estrogen production, and progesterone resistance play vital roles in endometriosis [55]. In endometriosis, the growth of endometrial tissue outside the uterus disrupts progesterone and estrogen signaling, resulting in decreased progesterone response and increased estrogen activity. This hormonal imbalance further leads to endometriotic symptoms, including inflammation, pelvic pain, and decreased endometrial receptivity for embryo implantation [38]. Hormonal inhibition has proven effective in reducing endometriosis recurrence and alleviating patient pain [56]. Our molecular docking result predicts that thymol competes with estrogen for binding to the estrogen receptor ESR1, thereby blocking relevant signaling pathways induced by the estrogen receptor, which can explain the role of thymol in this study. PGR is expressed both in stromal and epithelial cells of the uterus [57]. Epigenetic alterations, such as DNA methylation and histone modifications, on PGR are established during the progression of endometriosis [58]. Our findings demonstrate that thymol treatment leads to increased PGR expression at both the protein and mRNA levels, providing further evidence that thymol impacts endometriosis via rebalancing the estrogen and progesterone signaling. Thymol has been observed to directly inhibit estrogen-induced proliferation of endometriotic cells, evidenced by cell biology analyses and molecular docking results.

One of the main challenges for researchers is the uncertainty regarding the exact underlying mechanisms explaining the etiology and natural history of endometriosis. Sampson’s theory of retrograde menstruation is the most widely accepted hypothesis, describing how disruption to normal menstrual flow may result in endometriosis. Normally, the superficial (functional) endometrial layer is sloughed during menstruation (menses) to prepare the endometrium for the next menstrual cycle, resulting in vaginal bleeding for an average of 5 days [59]. In retrograde menstruation, shed tissue flows through the fallopian tubes, enters the pelvic cavity, and adheres to tissue in the pelvic cavity, leading to the formation of ectopic endometriosis lesions [60]. Studies suggest that endometriosis symptoms and lesion growth may vary with hormonal fluctuations. For example, estrogen plays a significant role in promoting lesion survival and inflammation in endometriotic tissue. This connection indicates that the menstrual or estrous cycle could impact disease progression and responses to treatment, especially in hormone-sensitive cases.

One limitation of this study is the relatively small sample size, which may reduce the statistical power and generalizability of the findings. A larger sample size would be beneficial to confirm the observed effects and ensure that the results are not due to random variation. Additionally, this study only utilized a single mouse model and did not incorporate human samples. While the mouse model provides valuable insights into the mechanisms of interest, the absence of human samples limits the applicability of the results to human physiology and pathology. Future studies could address these limitations by including a larger sample size and incorporating human-derived samples or clinical data to enhance the translational relevance of the findings. Furthermore, the strength of our study is that thymol has demonstrated potential as a therapeutic agent that does not adversely impact pregnancy, making it a promising candidate for use during pregnancy without compromising fetal development or maternal health. Thymol’s anti-inflammatory and antimicrobial properties can offer therapeutic benefits without disrupting the hormonal or physiological processes critical for maintaining pregnancy. This quality is particularly important in treating inflammatory or estrogen-sensitive conditions during pregnancy. Further research on in vivo human clinical trials would be valuable to validate thymol’s safety and efficacy in this context, ensuring it can be applied as a pregnancy-compatible treatment option.

## 4. Materials and Methods

### 4.1. Animal and Ethics Approval

The mice were procured from Guangdong Sijiajinda Biotechnology Co., Guangzhou, China, There were 30 mice included in this study, and the mice were provided food and water ad libitum and were maintained at a temperature of 22 ± 2 °C, a humidity of 50 ± 10%, and a light/dark cycle of 12 h. The Animal Care and Use Committee of South China Agricultural University approved all animal welfare procedures and investigations in this study (approval no. 2022b182, 9 November 2022).

### 4.2. Establishment of Murine Endometriotic Model

Seven-week-old female C57BL/6 mice were subcutaneously administered 17β-E2 (E8875, Sigma, St. Louis, MO, USA) at a dosage of 100 ng per mouse for three consecutive days to induce synchronous estrus. At the last injection day, the mice underwent surgical induction of endometriosis. The mice underwent anesthesia, followed by the excision and longitudinal dissection of one uterine horn. The dissected uterine horn was then fragmented into 2 × 2 mm sized pieces, which were subsequently sutured with peritoneum. Considering that the therapeutic doses of thymol were mostly set at 30 and 60 mg/kg in previous studies [61,62,63], the doses for our experiment were set at 30 and 60 mg/kg. Subsequent to endometriosis induction, the mice were divided into three groups, control (CTRL), Thymol 30 mg/kg, and Thymol 60 mg/kg, receiving oral administration of either saline or thymol (30 mg/kg/d or 60 mg/kg/d, T0501, Sigma), respectively. Each group consisted of ten mice and was evenly divided into estrus and diestrus according to the vaginal cytology on the last day of treatment. The mice were humanely killed following a treatment duration of three weeks to collect samples. Ectopic lesions were acquired and subsequently preserved in 4% paraformaldehyde (PFA) or stored in liquid nitrogen for further analysis.

### 4.3. Induction of Pregnancy and Treatment with Thymol

Female mice aged seven weeks were randomly divided into two groups and given either saline or thymol (60 mg/kg/d) orally, continued until sacrifice. These female mice were mated with fertile males of the same strain to induce conception on the 13th day of thymol administration and treatment continued for one more week until sacrifice on the 21st day. The appearance of vaginal plugs the subsequent day was deemed indicative of successful mating and recorded as 0.5 dpc (days post coitum). At 7.5 days post-coitum, mice (*n* = 3 per group) were euthanized and the implantation sites were harvested, either placed in liquid nitrogen or in PFA for other examination.

### 4.4. Histological Staining

Fixed uterine tissues were dehydrated using ethanol and xylene, embedded in paraffin, and cut into 5 µm thick pieces. H&E staining was accomplished on the sections using a previously described method after deparaffinization and rehydration [43].

For immunohistochemistry, as described previously [43], antigen retrieval was performed in sodium citrate solution, with heating by microwave after deparaffinization and rehydration. A 3% H_2_O_2_ solution inhibited the endogenous peroxidase. After that, samples were treated with 10% goat serum and left overnight at 4 °C to be exposed to primary antibodies. The next day, sections were incubated with a secondary antibody and avidin-HRP, followed by staining with a DAB substrate kit (Vector Laboratories, Burlingame, CA, USA). Finally, the sections were counter-stained with hematoxylin and imaged using a Leica microscopy imaging system (DM4000 B LED). Image J (2.9.0/1.54f, NIH, Bethesda, MD, USA) was used for all image analysis.

To perform immunofluorescence, the dewaxed paraffin sections were removed using sodium citrate. They were blocked with 10% horse serum, treated with 0.1% Triton X-100, and left for overnight incubation with primary antibodies at 4 °C. The slices were subsequently treated with Alexa Fluor 495-conjugated Donkey anti-Rat Immunoglobulin G (IgG) (Affineur, Düdingen, Switzerland), counter-stained with DAPI (Beyotime, Shanghai, China), and imaged using a confocal microscope (Leica, Nussloch, Germany). Table S1 contains a list of all the primary antibodies utilized in this investigation.

### 4.5. Cell Culture and Treatments

Ishikawa cells were cultured in DMEM/F12 (Sigma-Aldrich, St. Louis, MO, USA) supplemented with 1% penicillin/streptomycin (Gibco, Waltham, MA, USA) and 10% fetal bovine serum (Gibco). The cells were incubated at 37 °C in a humidified chamber with 5% CO_2_. For treatment, the cells were exposed to the following: vehicle (0.1% DMSO); 100 nM estrogen with or without 10 μM thymol, for 12 h.

### 4.6. Cell Viability Assay

Cell Counting Kit-8 (MCE) was used for cell viability assays. The procedures of the assays followed the manufacturer’s instructions. Cells were treated with five thymol concentrations (0, 10, 50, 100, and 250 μM). The dose of thymol was based on a previous study [61]. The cell viability rate was calculated as the optical density value of the sample/average optical density value of the control × 100%.

### 4.7. qPCR Assay

Total RNA was isolated using a Trizol RNA reagent (Takara, Berkeley, CA, USA) and reverse-transcribed into cDNA with the HiScript II Reverse Transcriptase kit (Vazyme, Nanjing, China). The ChamQTM Universal SYBR^®^ qPCR Master Mix (Vazyme) was employed to conduct qPCR on a Rotor-Gene Q system (Bio-Rad, Hercules, CA, USA). Melting curve analysis was employed to ascertain the specificity of PCR product amplification. The ΔΔCT method was used to calculate genetic expression. The mouse *Rpl19* or human *GAPDH* genes were used for normalization. qPCR was performed on both mouse tissue and Ishikawa cells. Table S2 shows all the primers used in this study.

### 4.8. Molecular Docking

The crystal structure of ESR1 was obtained from the RCSB Protein Data Bank (https://www.rcsb.org/, accessed on 13 November 2024) (PDB entry: 8DUC). The SDF structures of components were obtained from the PubChem site (https://pubchem.ncbi.nlm.nih.gov/, accessed on 13 November 2024). To obtain the docking−binding models, AutoDock Vina (https://vina.scripps.edu/, accessed on 13 November 2024) was used to perform the docking process of the flexible gland. Molecular docking was carried out using Vina in PyRx (https://pyrx.sourceforge.io/, accessed on 13 November 2024). For visual analysis, Pymol (https://pymol.org/2/, accessed on 13 November 2024) was employed, and the Discovery Studio 2020 Client (https://discover.3ds.com/discovery-studio-visualizer-download, accessed on 13 November 2024) was utilized for the analysis of the 2D model.

### 4.9. Statistical Analysis

All the analyses were performed using the GraphPad Prism 9.00 software. Unpaired Student’s *t*-tests were employed to compare two groups. One-way ANOVA with Tukey post hoc test was employed to evaluate the effects of thymol treatment on Ishikawa cells. Two-way ANOVA with Tukey post hoc test was employed to evaluate the effects and interaction of different thymol treatment groups and different estrous phases. The results are presented as means ± SEM. Statistical significance was set at *p* < 0.05.

## 5. Conclusions

In summary, the results demonstrated that thymol suppressed the proliferation of ectopic tissue in a mouse model of endometriosis due to its anti-inflammatory, anti-proliferative, and pro-apoptotic properties. Furthermore, thymol competes with estrogen for binding to ESR1, thereby preventing estrogen signaling that promotes proliferation in ectopic endometrial cells. Consequently, we propose thymol might be a novel natural product for the treatment of endometriosis. However, the efficacy of thymol in in vivo human trials still needs to be investigated further.

## Data Availability

The original contributions presented in the study are included in the article/Supplementary Materials, further inquiries can be directed to the corresponding authors.

## Supplementary Materials

The supporting information can be downloaded at: https://www.mdpi.com/article/10.3390/ijms252313150/s1.

## Abbreviations

MCs	Mesothelial cells
EDCs	Endocrine-disrupting chemicals
ESR1	Estrogen receptor 1
PGR	Progesterone receptor
BPA	Bisphenol A
MUC1	Recombinant Mucin 1
NSAIDs	Non-steroidal anti-inflammatory drugs
DES	Diethylstilbestrol
IFN-γ	Interferon-γ
TNF-α	Tumor Necrosis Factor-α
IL-1β	Interleukin-1β
IL-6	Interleukin-6
Ptgs2	Prostaglandin-Endoperoxide Synthase 2
COX2	Cyclooxygenase-2
CTRL	Control group
Ihh	Indian hedgehog
HAND2	Heart and neural crest derivatives-expressed transcript 2
HOXA10	Homeobox gene A10
AREG	Amphiregulin
MUC	Mucin
LFT	Lactotransferrin
F4/80	Mouse EGF-like module-containing mucin-like hormone receptor-like 1
Ly6G	Lymphocyte antigen 6complex, locus g
