# Supplementary material for: Thymol Impacts the Progression of Endometriosis by Disrupting Estrogen Signaling Pathways and Inflammatory Responses
Source: Int J Mol Sci. 2024 Dec 7;25(23):13150. doi: 10.3390/ijms252313150 (PMC11642495; doi:10.3390/ijms252313150)
Supplement: Supplementary file 1 [file ijms-25-13150-s001.zip › ijms-3294415-supplementary.pdf]

International Journal of *Molecular Sciences*

Article

Thymol impacts the progression of endometriosis by disrupting estrogen signaling pathways and inflammatory responses

Yu Zhang[1+], Aftab Shaukat[1+], Han Zhang[1], Yao-Feng Yang[1], Hui-Xia Li[1], Guang-Ya Li[1], Ying-Nan Liu[1], Chen Liang[1], Jin-Wen Kang[1], Shao-Chuan Li[1*], Ren-Wei Su[1,2*]

1. College of Veterinary Medicine, South China Agricultural University, Guangzhou 540642, China; 20231027017@stu.scau.edu.cn; dr.aftabshaukat@scau.edu.cn; 20222027044@stu.scau.edu.cn; 1271067857@stu.scau.edu.cn;lihuixia@stu.scau.edu.cn;13545080045@stu.scau.edu.cn;liuyingnan0902@163.com; 20221027009@stu.scau.edu.cn; jwkang2020@163.com; shaochuan@scau.edu.cn;
2. Key Laboratory of Animal Vaccine Development, Ministry of Agriculture, Guangzhou, China renweisu@scau.edu.cn

† Contributed equally to this work.

* Correspondence: renweisu@scau.edu.cn; shaochuan@scau.edu.cn

Citation: To be added by editorial staff during production.

Academic Editor: Firstname Lastname

Received: date
Revised: date
Accepted: date
Published: date

Copyright: © 2023 by the authors. Submitted for possible open access publication under the terms and conditions of the Creative Commons Attribution (CC BY) license (https://creativecommons.org/licenses/by/4.0/).

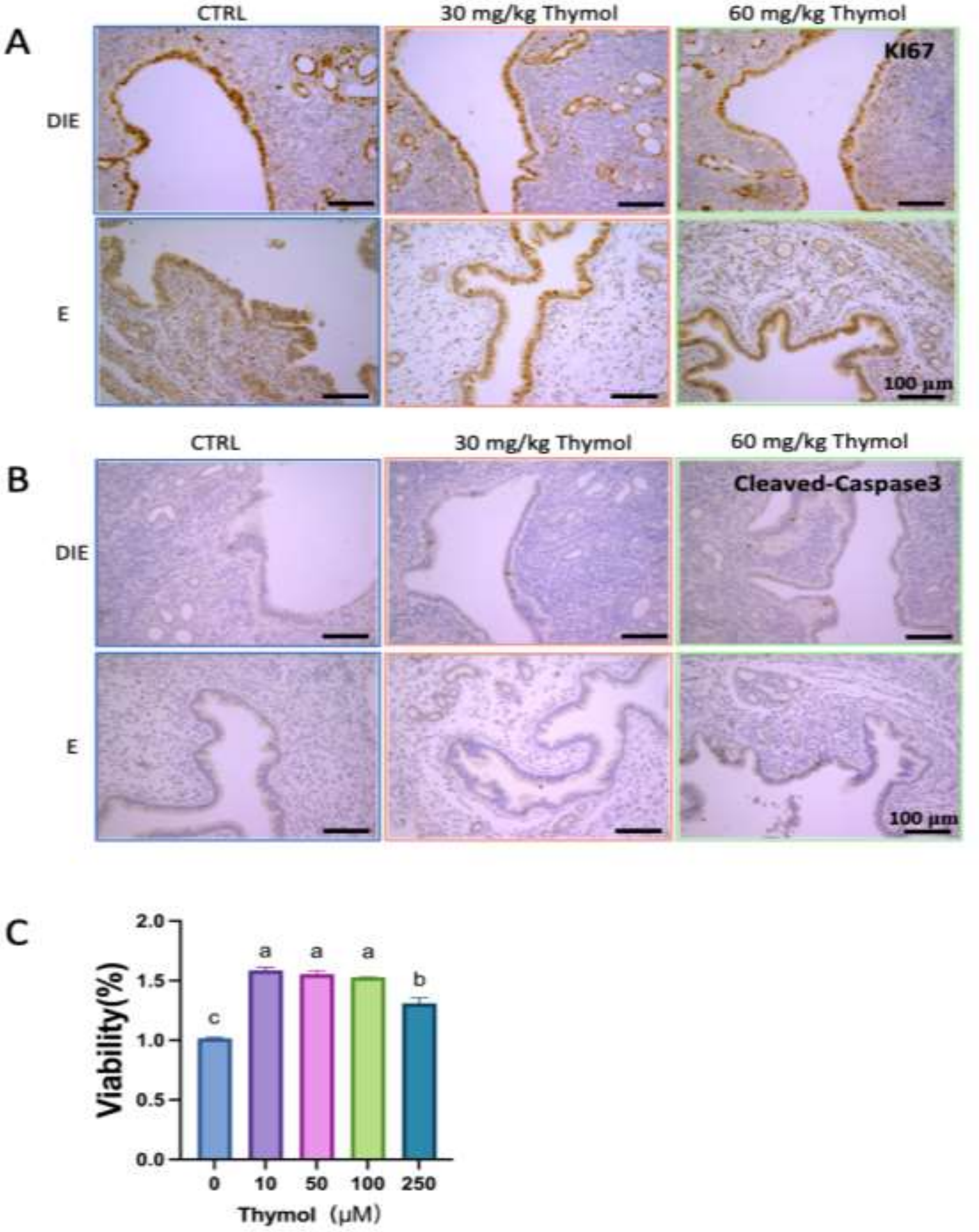

Figure S1. Thymol suppresses endometrial cell proliferation.
(**A**) Immunostaining of Ki67 in eutopic uteri from endometriosis mice, bar=100 µm. (**B**) Immunostaining of Cleaved-Caspase3 in eutopic uteri from endometriosis mice, bar=100 µm (**C**). Cell viability assay. DIE & E represent diestrus and estrus, respectively. Significant differences among several groups are displayed as different letters ($p < 0.05$).

Table S1. Primary antibodies.

Antibodies	Company	Application
Anit-Ki67	Axl-bio	IHC&IF (1:800)

Anit-Cleaved Caspase3	Cell Signaling Technology	IHC&IF (1:300)
Anti-F4/80	Abcam	IF (1:200)
Anti-Ly6G	Santa Cruz	IF (1:200)
Anti-MUC1	Cell Signaling Technology	IHC (1:200)
Anit-ERα	Abcam	IHC (1:5000)
Anit-PGR	Invitrogen	IHC (1:200)

IHC, immunohistochemistry; IF, immunofluorescence.

Table S2. Primer list for qPCR

Gene Symbol	Primer sequence（5' - 3'）
Mouse Rpl19	F: TCATGGAGCACATCCACAAGCTGA<br>R: CGCTTTCGTGCTTCCTTGGTCTTA
Mouse Esr1	F: GGAAGCTCCTGTTTGCTCCT<br>R: AACCGACTTGACGTAGCCAG
Mouse Esr2	F: AGTGCGTGGAAGGGATTCTG<br>R: GTCAGCTTCCGGCTACTCTC
Mouse Ltf	F: CAGCAGGATGTGATAGCCACAA<br>R: CACTGATCACACTTGCGCTTCT
Mouse Muc1	F: TTCCAACCCAGGACACCTAC<br>R: ATTACCTGCCGAAACCTCCT
Mouse Muc4	F: AATGTTCCTGCCTATACTGCC<br>R: TTGTATGGTTCCTGGGTCAC
Mouse Pgr	F: GTGGAAAAGCTATGTGCGCC<br>R: CAGTGGGGTTCAGGACCTTC
Mouse Ihh	F: CCCAACTACAATCCCGACATC<br>R: TCACCCGCAGTTTCACAC
Mouse Hoxa10	F: GAAAACAGTAAAGCTTCGCCG<br>R: GAAACTCCTTCTCCAGCTCC
Mouse Areg	F: AGATACATCGAGAACCTGGAGG<br>R: AGAGACAAAGATAGTGACAGCTAC
Mouse Hand2	F: TCGCCTACCTCATGGATCTGCT<br>R: TCTTGTCGTTGCTGCTCACTGT
Mouse Tnfa	F: AGGCACTCCCCCAAAAGATG<br>R: TGAGGGTCTGGGCCATAGAA
Mouse Ifng	F: AGGAACTGGCAAAAGGATGGT<br>R: GTTGCTGATGGCCTGATTGT
Mouse Il6	F: TCTTGGGACTGATGCTGGTGACA<br>R: AGCCTCCGACTTGTGAAGTGGTA
Mouse Il1b	F: TGGTGTGTGACGTTCCCATT<br>R: GCCCAAGGCCACAGGTATTT
Mouse Ki67	F: CCAGCTGCCTGTAGTGTCAA<br>R: CCATGTCTCAGCCTCACAGG
Mouse Bad	F: CTTGAGGAAGTCCGATCCCG<br>R: GCTCACTCGGCTCAAACTCT
Mouse Bcl2	F: TCGTCGCTACCGTCGTGACTT<br>R: TGAAGAGTTCCTCCACCACCGT
Mouse Prl8a2	F: AACCTCACTTCTCAGGGGCA<br>R: GAGCAGCCATTCTCTCCTGTT
Mouse Prl3c1	F: ATTGACTCAAGCACGCACCT

	R: GTGACGAGAAGAGGAAAGCAGA
Mouse Bmp2	F: ACACAGGGACACACCAACCAT<br>R: TGTGACCAGCTGTGTTCATCTTG
Mouse Wnt4	F: TCGTCTTCGCCGTGTTCT<br>R: CTGCACCTGCCTCTGGAT
Human GAPDH	F: GAAGGTGAAGGTCGGAGT<br>R: GATGGCAACAATATCCACTT
Human ESR1	F: CCTCCTCATCCTCTCCCACA<br>R: CTTTGGTCCGTCTCCTCCAC
Human LTF	F: GAGAGACTCCCCCATCCAGT<br>R: ACAGGTCGCAGTTTGTAGGG
Human MUC1	F: TGCTTACAGCTACCACAGCC<br>R: GCTGGGCACTGAACTTCTCT
Human MUC4	F: CCACAACCTCCCAGACCATC<br>R: CCCCATAGGGGAAGAGGGAA
Human PGR	F: GGAATGGGCTGTACCGAGAG<br>R: CGGCTCCTTTATCTCCCGAC
Human IHH	F: GCACCTGGAAGCAGTAACATGC<br>R: GGCAGCTATGGCTGCTAATGCA
Human HAND2	F: GGCAGAGATCAAGAAGACCGAC<br>R: CAGCGAGTTCAGGCGGTCCTT
